# A aplicação tópica de nanocápsulas de estrogênio na incisão cutânea melhora a Consolidação de fraturas em ratas osteoporóticas

**DOI:** 10.1055/s-0045-1809513

**Published:** 2025-06-14

**Authors:** Dalton Berri, Elcio Machinski, Conrado Auer Trentini, Paulo Vitor Farago, Adriana Yuriko Koga, Leandro Cavalcante Lipinski

**Affiliations:** 1 Departamento de Medicina, Universidade Estadual de Ponta Grossa, Ponta Grossa, PR, Brasil; 2 Departamento de Ciências Farmacêuticas, Universidade Estadual de Ponta Grossa, Ponta Grossa, PR, Brasil

**Keywords:** consolidação da fratura, estrogênios, nanotecnologia, osteoporose, estrogens, fracture healing, nanotechnology, osteoporosis

## Abstract

**Objetivo** O desafio de consolidação de fraturas osteoporóticas, particularmente exacerbadas pela deficiência de estrogênio pós-menopausa, ressalta a necessidade urgente de intervenções eficazes. Este estudo tem como objetivo avaliar o impacto do estrogênio administrado localmente via nanocápsulas na consolidação de fraturas osteoporóticas em ratas ovariectomizadas e analisar os efeitos sistêmicos deste hormônio usando o útero como órgão sentinela.

**Métodos** Quarenta e cinco animais foram submetidos a fraturas femorais padronizadas e divididos em três grupos: G1 (controle), G2 (estrogênio convencional) e G3 (nanocápsulas de estrogênio). O estrogênio foi aplicado topicamente na região da incisão da pele (área tricotomizada). A cicatrização da fratura foi avaliada 15 e 30 dias após a fratura por meio de análises radiográficas e histológicas. A histologia uterina analisou os efeitos sistêmicos.

**Resultados** Na análise radiográfica dos calos ósseos, G3 (8,75 ± 0,77 mm) exibiu formação de calo significativamente maior do que o grupo controle (7,18 ± 0,4 mm) no dia 15 e a análise histológica revelou aumento da formação de calo em G3 no dia 30, indicando um processo de cicatrização acelerado. Além disso, a análise histológica uterina no dia 30 mostrou uma redução na espessura endometrial em G3 (510.073 ± 54.705,11 μm) em comparação a G2 (623.729 ± 101.592 μm).

**Conclusão** Estes achados sugerem que nanocápsulas tópicas de estrogênio podem aumentar a formação de calos no tratamento de fraturas femorais osteoporóticas em ratas, potencialmente com menos efeitos sistêmicos.

## Abstract (en)

**Objective** The challenge of consolidating osteoporotic fractures, particularly exacerbated by postmenopausal estrogen deficiency, underscores the urgent need for effective interventions. This study aims to evaluate the impact of locally administered estrogen via nanocapsules on the consolidation of osteoporotic fractures in ovariectomized rats, while also assessing the systemic effects of this hormone, using the uterus as a sentinel organ.

**Methods** Forty-five animals underwent standardized femoral fractures and were divided into three groups: G1 (control), G2 (conventional estrogen), and G3 (estrogen nanocapsules). The estrogen was applied topically to the skin incision region (trichotomized area). Fracture healing was assessed at 15- and 30-days postfracture through radiographic and histological analyses, with uterine histology conducted to evaluate systemic effects.

**Results** In terms of radiographic analysis of callus formation, G3 (8.75 ± 0.77 mm) exhibited significantly higher callus formation than the control group (7.18 ± 0.4 mm) at day 15, with histological analysis revealing increased callus formation in G3 at day 30, indicating an accelerated healing process. Furthermore, uterine histological analysis at day 30 showed a reduction in endometrial thickness in G3 (510,073 ± 54,705.11 μm) compared with G2 (623,729 ± 101,592 μm).

**Conclusion** These findings suggest that topical estrogen nanocapsules may enhance callus formation in the treatment of osteoporotic femoral fractures in rats, potentially with fewer systemic effects.

## Introdução

As fraturas osteoporóticas representam um problema significativo de saúde pública e sua incidência aumenta a cada ano. 1 2 3 A maioria das estratégias terapêuticas se concentra na prevenção dessas lesões por meio do aumento da massa óssea, mas há menor ênfase no processo de consolidação do osso osteoporótico. 4 Além disso, a consolidação de fraturas osteoporóticas representa um desafio significativo para cirurgiões ortopédicos. 5

A deficiência de estrogênio, particularmente após a menopausa, é um importante fator de risco para a osteoporose. Este hormônio tem efeitos anabólicos e anticatabólicos, influenciando osteoblastos e osteoclastos no processo de remodelação óssea. 6 7 8 Além disso, ele é crucial para a regulação e formação do tecido cartilaginoso, afetando tanto a cartilagem de crescimento quanto as superfícies articulares. 9

Embora o papel do estrogênio no metabolismo ósseo e seu efeito protetor na densidade mineral óssea sejam bem conhecidos, 10 seu impacto na consolidação de fraturas ainda é obscuro. Poucos estudos avaliaram especificamente o papel desse hormônio no processo de cicatrização de fraturas osteoporóticas após a menopausa. 11 12 13 A redução dos níveis de estrogênio durante a menopausa contribui diretamente para um desequilíbrio na neoformação óssea, que pode prejudicar o processo de consolidação óssea em pacientes osteoporóticas. 14 15 Para atenuar esses efeitos, a aplicação tópica é vantajosa, pois evita o metabolismo hepático de primeira passagem, reduzindo, assim, a concentração necessária e minimizando os efeitos colaterais. 16

Os avanços na nanotecnologia permitem a manipulação de partículas para criação de veículos de transporte de medicamentos que visam órgãos específicos com segurança, melhorando a eficácia do transporte. 17 Os nanomateriais têm estruturas únicas com tamanho, forma e propriedades de superfície ajustáveis que impactam significativamente a absorção celular. 18 Em sistemas biológicos, partículas menores podem ser ideais para a absorção celular de compostos ativos. 19 20 Os nanossistemas podem ser benéficos na administração de medicamentos, melhorando a biodisponibilidade de ativos pouco solúveis, reduzindo os efeitos colaterais, liberando o fármaco de forma controlada e permitindo a administração de doses menores. 21

Neste estudo, avaliamos radiográfica e histologicamente o papel do estrogênio tópico aplicado à incisão cutânea, administrado tanto de forma convencional quanto por meio de nanocápsulas, no processo de consolidação da fratura femoral em ratas osteoporóticas e analisamos os efeitos sistêmicos desse hormônio usando o útero como órgão sentinela.

## Materiais e Métodos

### Desenvolvimento dos Produtos Farmacêuticos

Os fármacos foram desenvolvidos no Laboratório de Produção e Desenvolvimento de Medicamentos. Tanto as nanocápsulas quanto a formulação convencional apresentavam concentração de 17-β estradiol de 0,06%.

### Obtenção das Nanocápsulas Poliméricas Contendo 17-β Estradiol

As suspensões de nanocápsulas foram obtidas usando PCL (100 mg) dissolvido em acetona (30 mL) na presença de Span 80 (Croda International plc, Snaith, Reino Unido) com 0,077 g, 17-β estradiol (50 mg) e triglicerídeos de cadeia média (0,33 g). A solução foi agitada por 10 minutos. A fase aquosa foi preparada usando Tween 80 (Croda International plc) com 0,077 g e água destilada (53 mL). Em seguida, a fase orgânica foi lentamente adicionada à fase aquosa sob agitação magnética constante a 40°C. A nanoemulsão resultante foi agitada por 10 minutos. Em seguida, o solvente orgânico foi removido por evaporação sob pressão reduzida a 40°C, resultando em uma amostra concentrada (10 mL).

### Microscopia Eletrônica de Varredura por Canhão de Emissão de Campo (FEG-SEM)

A avaliação morfológica e de superfície da nanopartícula e da forma convencional foi realizada no microscópio eletrônico de varredura de canhão de emissão de campo modelo Mira 3 (TESCAN, Brno, República Tcheca). As amostras foram metalizadas com ouro usando um IC-50 Ion Coater (SHIMADZU, Kyoto, Japão). Micrografias eletrônicas foram obtidas usando uma tensão de aceleração de 15 kV e *software* específico (Electron Optical Design, Brno, República Tcheca).

### Dispersão Dinâmica da Luz e Microeletroforese por Doppler a Laser

O tamanho de partícula e o potencial zeta das nanopartículas (E2, PCLN e ZnON) foram determinados com instrumento Zetasizer Nano série ZS90 (Malvern Instruments, Worcestershire, Reino Unido) após preparação da amostra (1:500 V/V) em água ultrapura. As análises foram realizadas em triplicata.

### Modelo Animal

A pesquisa foi aprovada pelo Comitê de Ética em Uso de Animais (CEUA) sob o número de processo 0122368/2019. Todas as diretrizes institucionais e nacionais aplicáveis para o cuidado e uso de animais foram seguidas.

Foram 45 ratas Wistar fêmeas divididas em 3 grupos. O grupo 1 (G1), controle, tinha 15 ratas; o grupo 2 (G2) foi composto por 15 ratas tratadas com estrogênio em formulação convencional em uma concentração de 0,06% de 17 β-estradiol e o grupo 3 (G3) consistiu em 15 ratas tratadas com a mesma concentração de 17 β-estradiol, mas formulado como nanocápsulas.

### Técnica Anestésica

Nos procedimentos de ovariectomia e produção de fratura, os animais foram anestesiados com xilazina (10 mg/kg) e cetamina (90 mg/kg). Após a cirurgia, receberam uma dose única de citrato de fentanila (0,05 mg/kg) para controle da dor, seguido de dipirona (200 mg/kg) a cada 6 horas durante os primeiros 7 dias. Não foram utilizados anti-inflamatórios, para evitar uma possível interferência na avaliação da consolidação óssea. A dor pós-operatória foi monitorada pela avaliação do consumo de alimentos e água, bem como pela observação de mudanças comportamentais.

### Produção da Fratura

Após 40 semanas da ovariectomia, as ratas foram anestesiadas com a mesma técnica e submetidas a uma fratura do fêmur direito. A tricotomia e a antissepsia foram realizadas com iodo-povidona tópica (PVPI). Uma incisão de 2 cm foi feita na coxa lateral no membro posterior direito e estendida até o joelho. A dissecção foi realizada por planos e a patela foi retraída para expor os côndilos laterais. Um fio de Kirschner A de 1 mm de diâmetro foi inserido através dos côndilos até o trocanter maior e removido no dia 15. Após a fixação intramedular do fêmur, o músculo vasto lateral foi retraído, expondo a diáfise óssea. Uma fratura transversal foi feita na diáfise femoral usando um osteótomo de 5 mm. Após o procedimento, a fáscia muscular foi suturada com fio de poligalactina 2 a 0 absorvível e a pele foi suturada com fio de mononylon 3 a 0. Radiografias pós-operatórias imediatas confirmaram a fratura.

### Aplicação de Estrogênio

Os grupos 2 e 3 receberam aplicação diária de estrogênio tópico de acordo com seus respectivos grupos (pasta ou nanocápsulas na concentração de 0,06%) por 14 dias ao redor da ferida operatória (área já tricotomizada).

Após a aplicação do medicamento, cada grupo foi subdividido em 15 dias pós-fratura e 30 dias pós-fratura para eutanásia. Em seguida, os fêmures foram removidos e limpos para análise radiográfica e histológica.

### Avaliação Radiográfica

Um equipamento Lotus 630HF foi usado para obter radiografias de ambos os fêmures. O *software* VXvue 1.0.2.6pi (Viewwoks Co. Ltd., Anyang, South Korea) mediu o calo ósseo em seu maior diâmetro e o istmo femoral do fêmur contralateral. A medida absoluta do calo ósseo e a razão entre o calo e o istmo contralateral foram avaliadas.

### Análise Histológica

Os ossos, limpos de tecido muscular, foram armazenados em formaldeído a 10% e posteriormente descalcificados em solução de ácido etilenodiaminotetracético (EDTA) a 10%, trocada semanalmente por 2 meses. O segmento fraturado foi incluído em parafina, cortado longitudinalmente em espessura de 5 µm e corado com hematoxilina-eosina (HE). Após análise e seleção, os cortes histológicos foram fotografados com um microscópio Olympus DP72 (Evident Corp., Shinjuku-ku, Tóquio, Japão) utilizando o *software* cellSens Standard (Evident Corp.). As amostras foram submetidas às análises qualitativas e quantitativas. A escala numérica proposta por Huo et al. 22 foi aplicada de acordo com o estágio de consolidação observado em cada lâmina.

### Tecido Uterino

Após a eutanásia, todos os úteros foram coletados e fixados em formalina a 10%. O processamento histológico foi realizado e a amostra foi seccionada em micrótomo com espessura de 3 µm e corada com hematoxilina-rosina (HE). Os cortes histológicos foram fotografados usando um microscópio Olympus AX70 (Evident Corp.) com 20x de aumento, usando o programa T capture. A espessura das camadas do perimétrio, miométrio e endométrio foi medida com o *software* ImageJ após padronização da distância conhecida. A média e o desvio-padrão foram calculados para análise estatística subsequente.

### Análise Estatística

A avaliação estatística foi realizada no Statistical Package Social Sciences (SPSS, IBM Corp., Armonk, NY, USA) versão 20.0 por meio de análise de variância (ANOVA) para comparações múltiplas, seguida do teste de Tukey, com intervalo de confiança (IC) de 95% ( *p* ≤ 0,05).

## Resultados

### Processo de Produção das Nanocápsulas

O tamanho médio das nanopartículas de estrogênio foi de 191,96 ± 10,37 nm. A caracterização por microscopia eletrônica de varredura (MEV) é uma técnica que permite avaliar a influência das condições de síntese na morfologia das nanopartículas. Os resultados obtidos para estrogênio na forma convencional apresentaram partes grandes e irregulares. A MEV das nanopartículas de estradiol revelou formato esférico com superfície homogênea.

### Determinação da Concentração de Fármaco e Eficiência do Encapsulamento

A determinação da concentração do fármaco incorporado nas nanocápsulas e a eficiência de encapsulamento foram realizadas em triplicata utilizando o método previamente validado. As nanocápsulas obtidas pelo método de precipitação do polímero pré-formado apresentaram rendimentos superiores a 99%.

### Avaliação Radiográfica

Aos 15 dias, todos os animais já apresentavam sinais de formação de calo ósseo. Após 30 dias, todas as fraturas estavam radiologicamente consolidadas. As larguras do calo ósseo são mostradas na Tabela 1 .

**Tabela 1:** Média e desvio-padrão da largura do calo ósseo e da razão entre a largura do calo e do istmo contralateral aos 15 e 30 dias pós-fratura (mm)

	Dia 15		Dia 30	
	Largura	Razão	Largura	Razão
**G1**	7,18 ± 0,4 ^a^	2,04 ± 0,19 ^a^	8,3 ± 0,97 ^a^	2,4 ± 0,36 ^a^
**G2**	8,37 ± 1,4 ^ab^	2,43 ± 0,47 ^ab^	8,51 ± 0,9 ^a^	2,49 ± 0,2 ^a^
**G3**	8,75 ± 0,77 ^b^	2,54 ± 0,22 ^b^	8,53 ± 1,0 ^a^	2,56 ± 0,27 ^a^

**Abreviações:** G1, grupo 1; G2, grupo 2; G3, grupo 3 (tratamento com nanocápsulas de estrogênio). **Notas:** Os resultados são exibidos em mediana ± desvio padrão. Letras diferentes nas colunas denotam diferença significativa, *p* < 0,05.

**Fig. 1:**
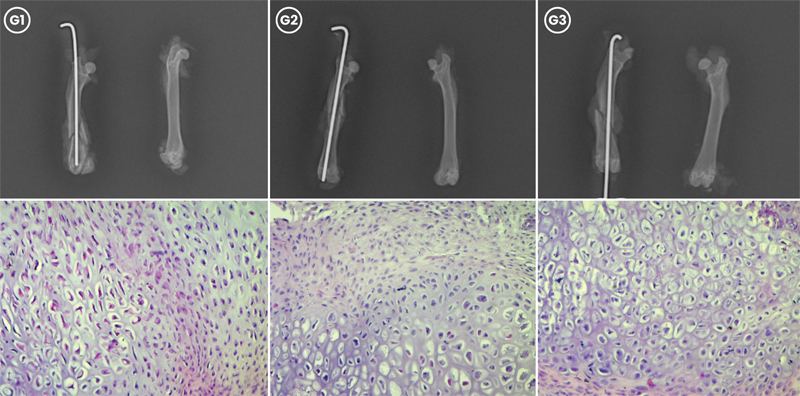
Avaliação radiográfica e histológica da consolidação da fratura femoral no G1 (controle), G2 (tratamento com estrogênio convencional) e G3 (tratamento com nanocápsulas de estrogênio) 15 dias após a fratura. (Acima) As radiografias mostram a formação de calo em G1, G2 e G3. (Abaixo) Cortes histológicos da formação de calo em cada grupo, corados com hematoxilina-eosina (aumento, 20x).

Na avaliação realizada 15 dias após a fratura, os animais do G3 apresentaram calo ósseo maior que os do G1 ( *p* < 0,05), enquanto os animais do G2 apresentaram calo ósseo estatisticamente igual ao G1 e G3, como demonstrado na Fig. 1 .

**Fig. 2:**
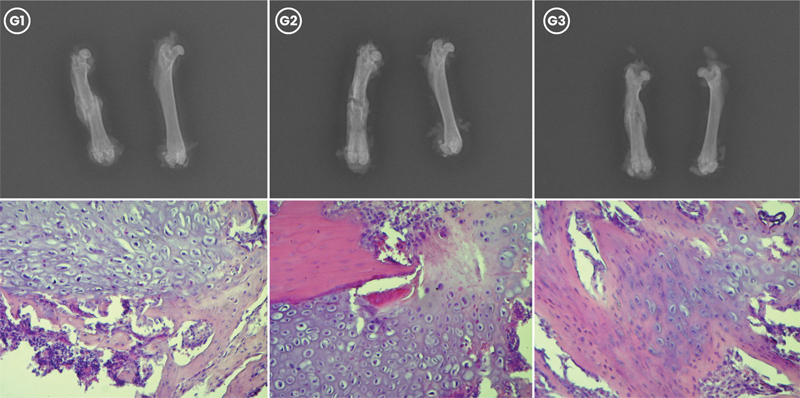
Avaliação radiográfica e histológica da consolidação da fratura femoral no G1 (controle), G2 (tratamento com estrogênio convencional) e G3 (tratamento com nanocápsulas de estrogênio) 30 dias após a fratura. (Acima) Radiografias mostrando fraturas consolidadas em G1, G2 e G3. (Abaixo) Cortes histológicos corados com hematoxilina-eosina, demonstrando formação de tecido ósseo e G3 apresentando maturação mais avançada do calo em comparação a G1 e G2 (aumento, 20x).

Os resultados da razão de tamanho mostram o mesmo resultado estatístico que o tamanho do calo ósseo, indicando a consistência dos achados ( Tabela 1 ). Aos 30 dias, não foi observada diferença significativa quanto ao tamanho e razão dos calos ósseos entre os grupos ( Fig. 2 ).

### Avaliação Histológica

A análise das lâminas revelou que. aos 15 dias ( Fig. 1 ), todas as ratas apresentavam predomínio de tecido cartilaginoso, sem diferença estatística entre os grupos ( Tabela 2 ). Aos 30 dias ( Fig. 2 ), G3 apresentou predomínio de tecido ósseo. enquanto os demais grupos ainda apresentavam maior quantidade de tecido cartilaginoso ( *p* < 0,05).

**Tabela 2:** Avaliação do escore histológico da maturação do calo ósseo proposto por Hou et al. 22 nos dias 15 e 30

	Dia 15	Dia 30
**G1**	5,28 ± 0,75 ^a^	7 ± 0,78 ^a^
**G2**	5,5 ± 1,2 ^a^	6,5 ± 0,79 ^a^
**G3**	5,8 ± 1,31 ^a^	7,83 ± 0,71 ^b^

**Abreviações:** G1, grupo 1; G2, grupo 2; G3, grupo 3 (tratamento com nanocápsulas de estrogênio). **Notas:** Os resultados são exibidos em mediana ± desvio padrão. Letras diferentes nas colunas denotam diferença significativa, *p* < 0,05.

Na avaliação do tecido uterino, observou-se que não houve diferença entre o G2 e G3 aos 15 dias, e os resultados foram significativamente maiores que os do G1 tanto no endométrio quanto no miométrio. Ao comparar os grupos aos 30 dias, G3 apresentou redução da espessura endometrial em relação a G2. Os resultados das medidas da espessura endometrial e miometrial são mostradas na Tabela 3 . Durante todo o experimento, não houve perdas amostrais.

**Tabela 3:** Avaliação da espessura do tecido uterino nos dias 15 e 30 (μm)

	Miométrio		Endométrio	
	Dia 15	Dia 30	Dia 15	Dia 30
**G1**	382.096 ± 75.017,84 ^a^	298.256 ± 53.430 ^a^	427.311 ± 97.735 ^a^	394.883 ± 79.977 ^a^
**G2**	458.528 ± 105.627,8 ^b^	440.251 ± 58.007 ^b^	783.537 ± 192.171 ^b^	623.729 ± 101.592 ^b^
**G3**	697.511 ± 98.439,72 ^b^	390.452 ± 76.422 ^b^	739.547 ± 131.672 ^b^	510.073 ± 54.705,11 ^c^

**Abreviações:** G1, grupo 1; G2, grupo 2; G3, grupo 3. **Notas:** Os resultados são exibidos em mediana ± desvio padrão. Letras diferentes nas colunas denotam diferença significativa, *p* < 0,05.

## Discussão

A ooforectomia realizada neste experimento foi suficiente para produzir um modelo de osteoporose e, consequentemente. alterar a formação do calo ósseo. Lill et al. 4 indicaram que esta doença diminui a formação de calo ósseo nos estágios iniciais de consolidação e a mineralização do calo nos estágios finais, com ratos osteoporóticos exibindo um calo 40% menor do que o grupo controle.

O estrogênio aumenta a diferenciação osteogênica das células-tronco mesenquimais e a maturação dos osteoblastos, favorecendo a formação óssea. Além disso, esse hormônio inibe a formação de osteoclastos e induz a apoptose dos osteoclastos, limitando a reabsorção óssea. Os receptores de estrogênio são altamente expressos em osteoblastos e osteócitos, gerando efeitos protetores no osso. 23

A deficiência de estrogênio altera a expressão dos genes alvo do estrogênio, induzindo a expressão de citocinas pró-inflamatórias, como interleucina (IL)-1, IL-6 e fator de necrose tumoral nos estágios iniciais do processo de consolidação, reduzindo a capacidade osteogênica e retardando a formação de calos. 24 Uma das ações do estrogênio é aumentar a liberação de fator de transformação do crescimento beta (TGF-β), o que estimula a produção de colágeno e proteoglicanos pelas células mesenquimais e osteoblastos, bem como a produção de fibronectina no tecido ósseo. 25

Beil et al. 7 analisaram o efeito do estrogênio em fraturas em ratos osteoporóticos e observaram aumento na formação de condrócitos nos estágios iniciais do processo de consolidação em animais tratados com pérolas de estrogênio, demonstrando que esse hormônio estimula a formação condral. No presente estudo, a administração deste hormônio na forma de nanocápsulas induziu maior produção de calo ósseo em 15 dias, como mostram as medidas. O estrogênio regula positivamente a função e a maturação dos condrócitos, influenciando a superfície articular e as epífises da placa de crescimento. 25 Esta observação, apoiada por Richmond et al., 26 é reforçada pelo achado de que o tamanho do calo ósseo foi significativamente maior nos grupos tratados com este hormônio, com G2 e G3 sendo iguais, mas apresentando um calo maior que o grupo controle.

Sabe-se que quanto mais cedo a fase inflamatória for superada e o processo de formação condral começar, mais cedo esse tecido irá se mineralizar e a fratura será reparada. 6 O papel do estrogênio se estende além da estimulação da condrogênese nos estágios iniciais, pois também influencia a consolidação do periósteo nos estágios finais da formação do calo. Beil et al. 7 observam altos níveis de calceína, um marcador de atividade osteoblástica, em ratas osteoporóticas tratadas com esse hormônio. Embora o presente estudo não tenha avaliado este marcador, podemos inferir que o resultado macroscópico encontrado se deve à melhor atividade celular influenciada pelo estrogênio.

A maior formação de calo ósseo observada no G2 e G3 demonstrou uma aceleração do processo atribuída ao estrogênio, com resultado estatisticamente melhor no grupo tratado com nanocápsulas. O uso de nanocápsulas melhora a ação local em 15 dias, pois o grupo tratado com nanocápsulas apresentou maior calo ósseo. Segundo Salimi et al., 27 o uso de estrogênio na forma de nanopartículas pode permitir o controle da taxa de liberação do ingrediente ativo, prolongando o efeito farmacológico no sítio da lesão.

Aos 30 dias, a avaliação radiográfica não mostrou diferença significativa no tamanho do calo ósseo entre os grupos ( *p* > 0,05). Apesar de G3 ter um calo ósseo maior aos 15 dias, não houve diferenças na maturação microscópica do calo entre os grupos. A avaliação histológica aos 30 dias revelou melhor maturação no G3. Essa maior qualidade é atribuída à estimulação precoce de células osteocondrogênicas pelas nanocápsulas de estrogênio. 28

O G3 apresentou melhor formação de calo ósseo em comparação aos outros grupos, com calo maior em 15 dias e melhor qualidade em 30 dias, atribuído à melhor ação e permeação do fármaco. Kaur et al. 29 observaram maior permeabilidade com formulações de nanocápsulas em ratos osteoporóticos, apoiando nossos achados de que elas melhoram significativamente a difusão do fármaco para o sítio da fratura em comparação aos métodos convencionais.

A avaliação do útero como órgão sentinela em nosso estudo monitorou o efeito dos tratamentos no tecido endometrial. Aos 15 dias, ambos apresentaram aumento do tecido endometrial e miometrial em comparação ao grupo não tratado. Entretanto, aos 30 dias, o tamanho endometrial em G3 foi menor em comparação ao G2. Essa observação reforça os resultados encontrados no processo de formação do calo ósseo. Os fármacos nanoencapsulados atingem melhor o sítio de ação, deixando uma quantidade menor para efeitos sistêmicos. Silva et al. 30 não observaram efeitos sistêmicos do tratamento tópico com estrogênio, discordando do que foi encontrado em nosso estudo.

### Limitações do Estudo

Este estudo encontrou várias limitações. Primeiro, não avaliamos a concentração efetiva mínima de nanocápsulas de estrogênio especificamente adaptadas para esta aplicação. Além disso, embora nossa investigação tenha se concentrado principalmente nos efeitos locais do hormônio na cicatrização óssea e suas possíveis implicações sistêmicas no tecido uterino, reconhecemos que a avaliação de outras implicações sistêmicas além do tecido uterino poderia oferecer uma compreensão mais abrangente de seu impacto geral. Por fim, não exploramos concentrações alternativas na administração de estrogênio, o que poderia revelar efeitos dependentes da dose e dar outros caminhos para otimização em estudos futuros.

## Conclusão

Considerando os resultados deste estudo, o estrogênio acelerou o processo de consolidação da fratura em ratas osteoporóticas, principalmente pela aceleração da fase condral e culminando em melhor matriz óssea em 30 dias. A opção de estrogênio em nanocápsulas obteve um resultado melhor do que a administração convencional. É importante ressaltar que os efeitos sistêmicos, avaliados por meio da análise do tecido uterino, revelaram uma redução significativa na espessura endometrial no grupo tratado com nanocápsulas em comparação aos tratados com estrogênio convencional, indicando a possibilidade de menos efeitos colaterais sistêmicos.

Parece haver espaço para o uso de estrogênio local em nanocápsulas para o tratamento de fraturas osteoporóticas após a menopausa. Uma concentração desse hormônio no sítio da fratura estimula e acelera o processo de formação do calo ósseo, evitando, assim, complicações inerentes a esse tipo de fratura. A dose administrada e a redução das repercussões sistêmicas devem ser analisadas em maior profundidade em estudos subsequentes.
